# Practical methods for handling human periodontal ligament stem cells in serum-free and serum-containing culture conditions under hypoxia: implications for regenerative medicine

**DOI:** 10.1007/s13577-017-0161-2

**Published:** 2017-02-06

**Authors:** Dai Murabayashi, Mai Mochizuki, Yuichi Tamaki, Taka Nakahara

**Affiliations:** Department of Developmental and Regenerative Dentistry, School of Life Dentistry at Tokyo, The Nippon Dental University, 1-9-20 Fujimi, Chiyoda-ku, Tokyo, 102-8159 Japan

**Keywords:** Periodontal ligament, Mesenchymal stem cell, Serum-free culture, Hypoxia, Cytotoxic susceptibility

## Abstract

Stem cell-based therapies depend on the reliable expansion of patient-derived mesenchymal stem cells (MSCs) in vitro. The supplementation of cell culture media with serum is associated with several risks; accordingly, serum-free media are commercially available for cell culture. Furthermore, hypoxia is known to accelerate the expansion of MSCs. The present study aimed to characterize the properties of periodontal ligament-derived MSCs (PDLSCs) cultivated in serum-free and serum-containing media, under hypoxic and normoxic conditions. Cell growth, gene and protein expression, cytodifferentiation potential, genomic stability, cytotoxic response, and in vivo hard tissue generation of PDLSCs were examined. Our findings indicated that cultivation in serum-free medium does not affect the MSC phenotype or chromosomal stability of PDLSCs. PDLSCs expanded in serum-free medium exhibited more active growth than in fetal bovine serum-containing medium. We found that hypoxia does not alter the cell growth of PDLSCs under serum-free conditions, but inhibits their osteogenic and adipogenic cytodifferentiation while enabling maintenance of their multidifferentiation potential regardless of the presence of serum. PDLSCs expanded in serum-free medium were found to retain common MSC characteristics, including the capacity for hard tissue formation in vivo. However, PDLSCs cultured in serum-free culture conditions were more susceptible to damage following exposure to extrinsic cytotoxic stimuli than those cultured in medium supplemented with serum, suggesting that serum-free culture conditions do not exert protective effects against cytotoxicity on PDLSC cultures. The present work provides a comparative evaluation of cell culture in serum-free and serum-containing media, under hypoxic and normoxic conditions, for applications in regenerative medicine.

**Electronic supplementary material:**

The online version of this article (doi:10.1007/s13577-017-0161-2) contains supplementary material, which is available to authorized users.

## Introduction

Mesenchymal stem cells (MSCs) comprise a multipotent cell population capable of extensive proliferation and differentiation into a variety of cell types including osteoblasts, adipocytes, chondrocytes, myoblasts, and neurons [1, 2]. MSCs can be efficiently isolated from several tissues including bone marrow, adipose tissue, and umbilical cord (Wharton’s jelly) [3–5]. In addition, teeth, which are usually discarded during common extraction procedures, represent a source of multiple types of dental tissue-derived MSCs [6]. We previously reported the successful isolation and characterization of four types of human dental MSCs derived from dental pulp, periodontal ligament (PDL), apical papilla, and dental follicle, which were all collected from extracted mature or immature wisdom teeth [7]. Notably, these dental MSCs exhibited greater proliferative ability than iliac bone marrow-derived MSCs along with similar multidifferentiation potential and gene/protein expression profiles. Furthermore, we and other researchers have reported that PDL-derived MSCs (PDLSCs) in particular are beneficial for stem cell therapy in preclinical trials with large animals [8–10].

In conventional in vitro MSC culture, supplementation of culture media with fetal bovine serum (FBS) is essential for facilitating various cell behaviors such as cell attachment to culture dishes, cell growth, and cytodifferentiation. However, the use of FBS involves potential risks such as xenogeneic immune response and pathogenic infection [11, 12]; accordingly, several FBS-free culture media for expanding MSCs have been developed for commercial use [13–15]. In addition, low oxygen tension in the cell culture environment has been shown to exert a positive effect on cell growth and proliferation, enabling enhanced numbers of MSCs to be obtained in vitro culture [16, 17]. Hypoxic culture conditions, therefore, represent a potential alternative method for substantially accelerating MSC expansion.

The cellular phenotypes and behaviors of dental MSCs including PDLSCs, such as cell growth, gene/protein expression, cytodifferentiation potential, genomic stability, cytotoxic reactions, and in vivo tissue generation under serum-free and hypoxic culture conditions, remain poorly understood. Here, we aimed to characterize the stem cell properties of PDLSCs under serum-free and hypoxic culture conditions, and to establish practical cell culture methods for obtaining cells for clinical applications and cell-based therapies.

## Materials and methods

### Cell isolation and cell culture

Normal human third molars were obtained from healthy patients aged 27–32 years undergoing tooth extraction. Cell isolation and culture of PDLSCs were performed according to our previous study [7]. Briefly, PDL tissue was gently scraped from the middle third of the tooth root using a sterilized razor blade. The collected tissues were minced and then digested in a solution containing 3 mg/mL collagenase type I (Merck KGaA, Darmstadt, Germany) and 4 mg/mL dispase (Wako Pure Chemical Industries, Osaka, Japan) for 1 h at 37 °C. The cells were passed through a 70-µm cell strainer; the resulting single-cell suspensions (1 × 10^5^ cells/dish) were seeded into 100-mm dishes and cultured in Dulbecco’s modified Eagle’s medium/Ham’s nutrient mixture F12 (Thermo Fisher Scientific, Waltham, MA, USA) supplemented with 15% FBS (Lot No: 027K0361, Merck KGaA), 100 µM glutamate (GlutaMAX I; Thermo Fisher Scientific), 0.1% MEM non-essential amino acids (Thermo Fisher Scientific), 50 U/mL penicillin, 50 µg/mL streptomycin, and 0.25 mg/mL amphotericin B (Fungizone). This recipe was designated as FBS-containing medium (FCM). Upon reaching 70–80% confluence, cells were detached using calcium- and magnesium-free phosphate-buffered saline (PBS) containing 0.25% trypsin and 0.02% ethylenediaminetetraacetic acid (EDTA). Then, the detached cells were subcultured at a 1:3 split ratio. The cells were cultured in either FCM or FBS (serum)-free culture medium supplemented with MSC-T4 supplement [18] [Cell Science Technology Institute (CSTI), Sendai, Japan]; this medium was designated as SFM. For SFM cell culture, culture dishes/plates were pre-coated with MSC-T4 coating reagent (CSTI) [18].

Cell cultures were maintained at 37 °C in 4.7% CO_2_ humidified air, which was designated as the normoxic condition (ambient O_2_ concentration). For hypoxic cell culture, cells were maintained in the same manner, except that 3% O_2_, balanced with N_2_, was utilized via an O_2_- and CO_2_-controlled multi-gas incubator (MCO-5M; Sanyo, Osaka, Japan). Culture media were changed every 3–4 days. Cells at passage 3 or 4 were used for the subsequent experiments. For each experiment, cells derived from at least three different individuals were used.

### Morphological analysis of cultured cells

The cell morphology of PDLSCs cultured in SFM or FCM was evaluated according to our previous study [7], with minor modifications. Briefly, 3000 cells were randomly selected in subconfluent cultures. Spindle-shaped cells, which were defined as cells that extend a thin process at least three times longer than their cell body, were counted via microscopic observation. The results were expressed as the percentage of spindle-shaped cells with long cell processes.

### Cell growth evaluation

Cells (1 × 10^4^ cells/well) were seeded into 24-multiwell plates and cultured with SFM or FCM under normoxic or hypoxic conditions. Cultured cells were collected by adding trypsin–EDTA and counted in triplicate using a hemocytometer every 48 h for 14 days. The population doubling time (PDT) was calculated using the following formula: (*t* − *t*_0_)log_2_/log(*N* − *N*_0_), where *t* is time (hours), *N* is the number of harvested cells, and *N*_0_ is the number of cells in the inoculum.

### Flow cytometry for cell cycle analysis

Cells collected by the trypsin–EDTA treatment were fixed with cold 70% ethanol for 2 h at −20 °C. After washing with PBS, the fixed cells were stained with propidium iodide (PI) for 30 min at room temperature (RT). The PI-elicited fluorescence was measured using a Guava™ EasyCyte HT instrument and Guava™ cell cycle software version 2.7 (Guava Technologies, Hayward, CA, USA).

### Flow cytometry for cell-surface marker analysis

After trypsinizing cultured cells, the cells were fixed in cold methanol for 10 min at 4 °C. Cells were then incubated for 1 h at RT with an appropriate amount of the following antibodies: fluorescein isothiocyanate (FITC)-conjugated antibodies against CD14, CD90, and CD105 (Becton–Dickinson, San Jose, CA, USA); CD34 and CD44 (Beckman Coulter, Fullerton, CA, USA); and phycoerythrin-conjugated mouse immunoglobulin (Ig) G1 antibody (Becton–Dickinson). The expression profiles were analyzed using a Guava™ EasyCyte HT and Guava™ Express Plus (version 2.7) software (Merck KGaA).

### Reverse-transcription polymerase chain reaction (RT-PCR)

RT-PCR was performed as previously described [19]. The specific primer sequences and PCR amplification conditions are listed in Table 1.

**Table 1:** Primer sequences and amplification conditions for RT-PCR analysis

Gene	Primer sequences, 5′ to 3′	Product size (bp)	Annealing temp. ( °C)	PCR cycles	GenBank accession number
*Vimentin*	F: GGGACCTCTACGAGGAGGAG	200	55	35	NM_003380
	R: CGCATTGTCAACATCCTGTC				
*Collagen I*	F: CCAAATCTGTCTCCCCAGAA	214	55	35	NM_000088
	R: TCAAAAACGAAGGGGAGATG				
*Runx2*	F: CCCCACGACAACCGCACCAT	292	59	35	NM_001015051
	R: GTCCACTCCGGCCCACAAATC				
*Nestin*	F: AACAGCGACGGAGGTCTCTA	220	55	35	NM_006617
	R: TTCTCTTGTCCCGCAGACTT				
*Nanog*	F: ACCTTCCAATGTGGAGCAAC	199	55	35	NM_024865
	R: GAATTTGGCTGGAACTGCAT				
*Oct3/4*	F: GACAGGGGGAGGGGAGGAGCTAGG	144	60	35	NM_001173531
	R: CTTCCCTCCAACCAGTTGCCCCAAAC				
*Sox2*	F: AACCCCAAGATGCACAACTC	152	60	40	NM_003106
	R: CGGGGCCGGTATTTATAATC				
*GAPDH*	F: GAGTCAACGGATTTGGTCGT	238	55	35	NM_002046
	R: TTGATTTTGGAGGGATCTCG				

*Collagen I* Collagen type I alpha 1 chain, *Runx2* Runt-related transcription factor 2, *Nanog* Nanog homeobox, *Oct3/4* POU class 5 homeobox 1 (POU5F1), *Sox2* SRY-box 2, *GAPDH* glyceraldehyde-3-phosphate dehydrogenase

### In vitro multilineage differentiation

In vitro osteogenic- and adipogenic differentiation experiments in PDLSCs were performed according to our previous study [7]. For chondrogenic differentiation, a pelleted micromass of 1 × 10^5^ cells was formed by centrifugation at 430×*g* for 5 min and then cultured with α-MEM containing 10% FBS, 10 ng/mL transforming growth factor-β1 (PeproTech, Rocky Hill, NJ, USA), 50 mM _L_-ascorbic acid 2-phosphate magnesium salt *n*-hydrate, and 50 mM ITS + 1 (Merck KGaA) for 4 weeks. After the differentiation, pellets were fixed with 10% neutral buffered formalin, embedded in paraffin, and cut into sections of 5-μm thickness for histological analysis. Chondrogenic differentiation was evaluated by staining with solutions of 1% Alcian blue and 0.1% Safranin O, and by immunohistochemistry using a rabbit polyclonal anti-type-II collagen antibody (1:50; Santa Cruz Biotechnology, Dallas, TX, USA).

### Immunocytofluorescence

Cultured cells were plated at 1 × 10^5^ cells/well in four-well chamber slides and fixed with cold methanol for 10 min at −30 °C. After washing with PBS, cells were incubated in Blocking One Histo (Nacalai Tesque, Kyoto, Japan) for 10 min at RT. Cultures were incubated with the following primary antibodies at 4 °C overnight: rabbit polyclonal anti-nestin, rabbit polyclonal anti-neurofilament-200, and chicken polyclonal anti-βIII-tubulin (all diluted 1:1000; Merck KGaA). After washing with PBS, the samples were incubated with the following secondary antibodies for 30 min at RT in the dark: Alexa Fluor 488-conjugated goat anti-mouse IgG, Alexa Fluor 488-conjugated donkey anti-rabbit IgG, and Alexa Fluor 488-conjugated goat anti-chicken IgY (all diluted 1:1000; Thermo Fisher Scientific). The samples were washed with PBS three times for 5 min each and mounted with Vectashield mounting medium containing 4′,6-diamidino-2-phenylindole (Vector Laboratories, Burlingame, CA, USA). For negative controls, the primary antibody was omitted during immunostaining. Images were obtained using a Biorevo BZ-9000 microscope (Keyence, Osaka, Japan).

### Karyotype analysis

Karyotype G-band analysis was performed as previously described [20].

### Assessment of cytotoxic stimuli-induced cell viability and cell damage

To investigate whether SFM or FCM cultures are affected by extrinsic cytotoxicity, cultured PDLSCs were subjected to treatment with extrinsic stimuli via exposure to staurosporine, hydrogen peroxide (H_2_O_2_), or ultraviolet radiation (UV), and cellular stress/damage was detected. Cells were seeded into 24-multiwell plates (1 × 10^5^ cells/well) and treated with the following: 1 µM staurosporine (Wako Pure Chemical Industries) or 1 µM H_2_O_2_ (Wako Pure Chemical Industries) for 12 h at 37 °C. Alternatively, cells were seeded into 60-mm dishes (1 × 10^6^ cells/dish) and irradiated with UV light using an UV transilluminator (UVP, Upland, CA) for 30 min at RT.

### Detection of cell viability using an 3-(4,5-dimethylthiazol-2-yl)-2,5-diphenyltetrazolium bromide (MTT) assay

Cell viability of the PDL-derived cells exposed to cytotoxic stimuli was assessed via the MTT colorimetric assay. Following treatment with staurosporine, H_2_O_2_, or UV, 50 μL MTT-tetrazolium salts (5 mg/mL) (Merck KGaA) in PBS was added to each well in 24-multiwell plates. After 2 h incubation at 37 °C, the formazan crystals were dissolved by adding 300 μL isopropanol. Absorbance of the supernatant fluid was measured at 570 nm wavelength using a Corona Grating Microplate Reader (Hitachi High-Technologies). The cell viability rate (%) was calculated as the sample optical density (OD) divided by control OD × 100.

### Detection of cellular stress/damage by flow cytometric analysis with Annexin V/PI staining

Cellular stress/damage in the aforementioned cytotoxic stimuli-treated PDLSCs was determined by flow cytometric analysis using an Annexin V-FITC/PI system (Guava Technologies), according to the manufacturer’s instructions. Briefly, cells exposed to each stimulus were incubated with 100 μL Guava Nexin Reagent for 20 min at RT in the dark and analyzed using the Guava™ EasyCyte HT and Guava™ InCyte System (version 2.7). Annexin V-positivity and PI-negativity indicated the presence of early apoptotic cells, and Annexin V- and PI-double positivity indicated the presence of late apoptotic cells. In this study, both classes of fractionated cells were considered to represent damaged cells. The damaged cell rate (%) was calculated as the number of damaged cells divided by the number of total cells tested × 100.

### In vivo transplantation and histological evaluation

To examine the hard tissue formation capacity in vivo, PDLSCs expanded using SFM or FCM were subcutaneously transplanted under the dorsal skin of 6-week-old female nude mice (BALB/C-nu/nu; Nihon Clea, Tokyo, Japan). The cell/scaffold construct consisted of a mixture of approximately 1  ×  10^6^ cells and 400 µL collagen gel (Nitta Gelatin, Osaka, Japan) with 40 mg hydroxyapatite (HA)/β-TCP-scaffold (Ceraform^®^; NGK Spark Plug, Aichi, Japan). All transplants were collected for histological analysis at 16 weeks post-transplantation (*n* = 6 per group).

Histological and immunohistochemical examinations were performed as previously described [21]. Primary antibodies were used as follows: mouse monoclonal anti-human-specific vimentin (1:10,000; Merck KGaA), rabbit polyclonal anti-osteopontin (OPN; 1:1000; Abcam, Cambridge, MA, USA), and rabbit polyclonal anti-cementum attachment protein (CAP; 1:100; LifeSpan Biosciences, Seattle, WA, USA). For negative controls, the primary antibody was omitted during immunostaining.

### Statistical analysis

Quantitative data were obtained from three independent experiments. All samples were analyzed in triplicate and the data were expressed as the mean ± standard deviation. Representative results were indicated in each experiment. Statistical analysis was performed using two-way ANOVA with Bonferroni’s correction or Fisher’s Exact test. A probability of *P* < 0.01 was considered to indicate a statistically significant difference. Data were analyzed using IBM SPSS Statistics software (version 23.0; IBM Japan, Tokyo, Japan).

## Results and discussion

### PDLSCs cultured in SFM exhibit more active cell growth than those cultured in FCM under normoxic culture conditions

**Fig. 1:**
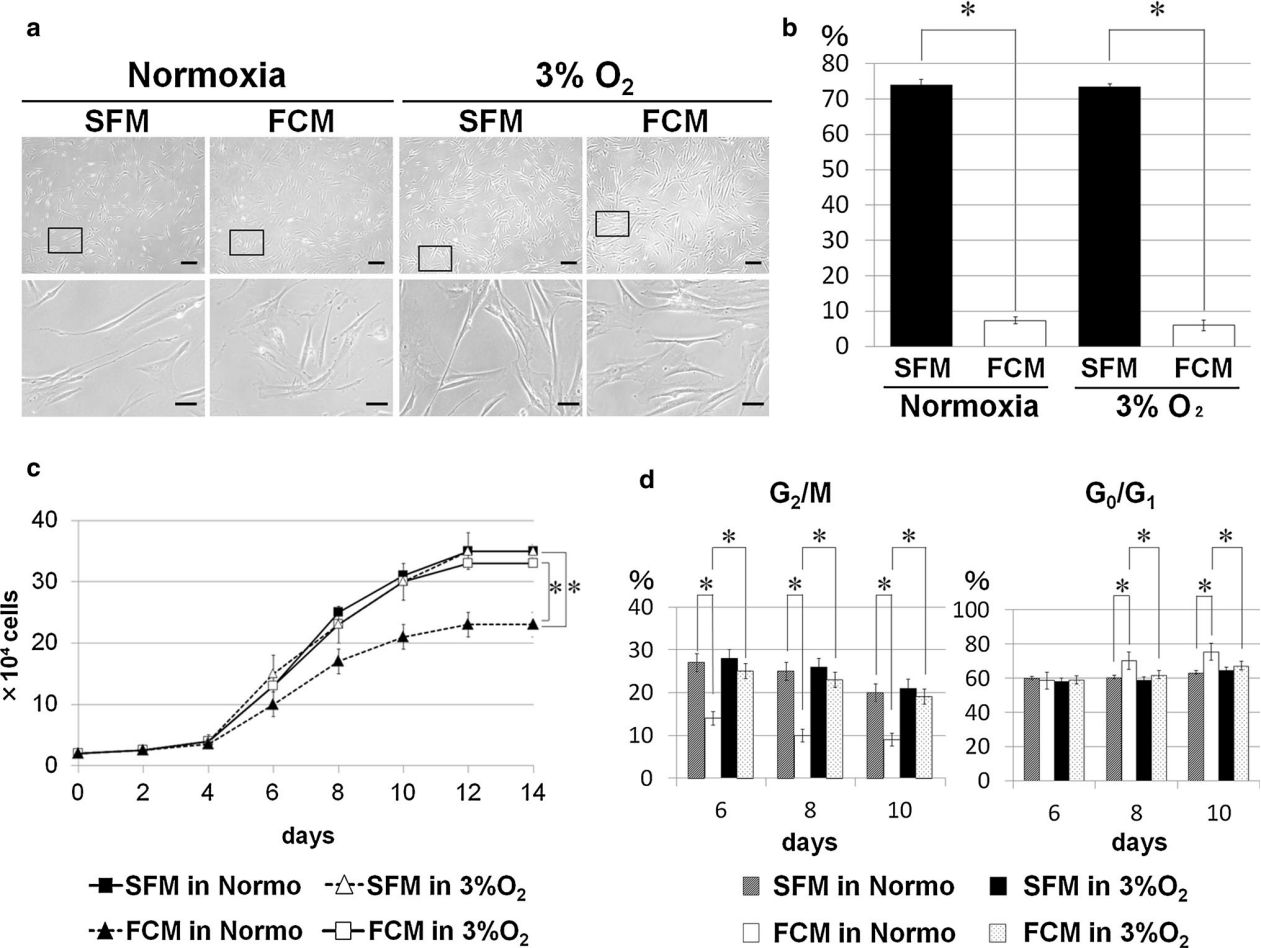
Morphological evaluation and cell growth characteristics of PDLSCs cultured in the presence or absence of FBS, under either normoxic or hypoxic culture conditions. **a** Phase-contrast images of PDLSCs cultured in serum-free medium (SFM) or FBS-containing medium (FCM) under normoxic (normo) or hypoxic (3% O_2_) culture conditions; the *lower panels* show enlarged views indicated by the *boxed area in the upper panels*. All the PDLSC cultures primarily showed fibroblastic cell morphology when cultivated in SFM and FCM under normoxic and hypoxic conditions. In particular, SFM-cultured PDLSCs exhibited a more spindle-shaped morphology under hypoxic conditions. *Scale bars* 200 µm (*upper panels*); 50 µm (*lower panels*). **b** Morphological evaluation of the length of cell processes of PDLSCs cultured in SFM or FCM based on hypoxia (*P < 0.01). The cell rate (%) indicates the number of cells with longer processes divided by the number of total cells examined. **c** Growth curve analysis of SFM- and FCM-cultured PDLSCs under hypoxic and normoxic conditions at 14 days post-seeding; FCM-cells under hypoxia (*open squares*) and SFM-cells under normoxia (*closed squares*) showed a significantly greater growth rate than FCM-cells under normoxia (*closed triangles*) at day 8 or later in culture (*P < 0.01). Notably, no significant difference was found between the growth of SFM cells under normoxic (*closed squares*) and hypoxic (*open triangles*) culture conditions. Further, there was no significant difference in cell growth between SFM cells (*open triangles*) and FCM cells (*open squares*) under hypoxic conditions during the culture period. **d** Flow cytometric analysis for cell cycle status indicated that a greater cell growth of FCM cells under hypoxia and SFM cells under normoxia than FCM cells under normoxia, and that the proportion of cells in G2/M phase was significantly higher, and those in the G0/G1 phase significantly lower, among FCM cells under hypoxia and SFM cells under normoxia relative to FCM cells under normoxia during the logarithmic growth phase in culture period (*P < 0.01)

SFM- and FCM-cultured PDLSCs showed typical fibroblastic cell morphology under normoxic culture conditions (Fig. 1a) although SFM-cultured cells exhibited significantly greater spindle-shaped morphology, with longer cell processes, than FCM-cultured cells (Fig. 1b). Similar findings are described in some previous reports of bone marrow-derived MSCs cultured under serum-free culture conditions [14, 22]. The cell growth of SFM-cultured PDLSCs under normoxic conditions was significantly higher than that of FCM-cultured cells at day 8 or later in culture (Fig. 1c). Flow cytometric analysis of cell cycle status during the logarithmic growth phase also indicated active SFM cell division (Fig. 1d), consistent with previous reports [15, 23].

**Fig. 2:**
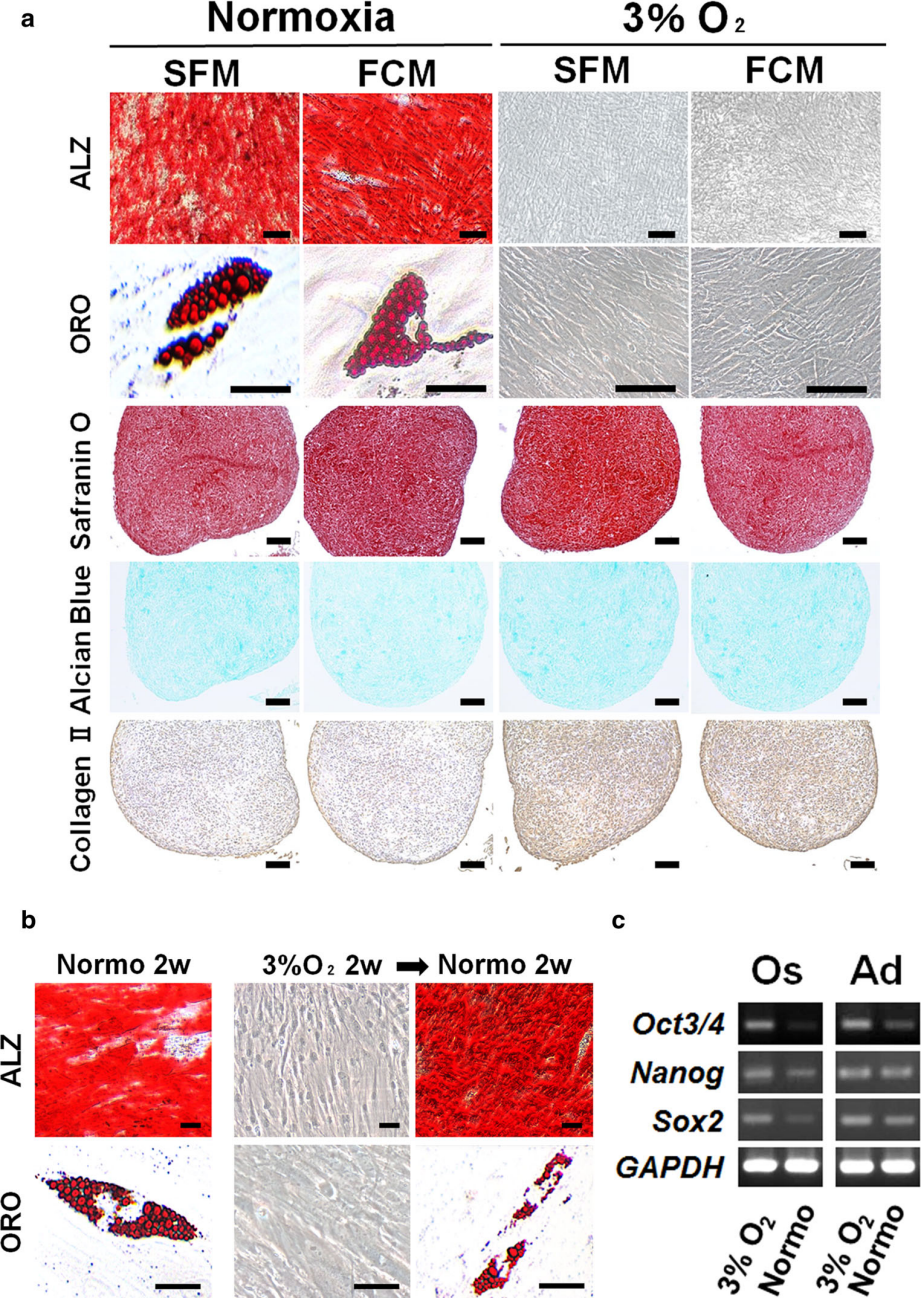
In vitro multilineage differentiation potential of PDLSCs cultured in the presence or absence of FBS under hypoxic or normoxic culture conditions. **a** Alizarin red S (ALZ)- and oil red O (ORO)-staining indicate mineralized deposits and lipid clusters, respectively, in SFM- or FCM-cells under normoxic conditions 4 weeks after culturing in the respective cytodifferentiation medium. In contrast, no positive staining was observed in either SFM- or FCM-cells under hypoxic conditions (3% O_2_). Chondrogenic differentiation was achieved in all the PDLSC-pellet cultures after 4 weeks (Safranin O and Alcian blue); particularly, cytodifferentiation was enhanced under hypoxic culture conditions, as indicated by the intense immunoreactivity for type II collagen (Collagen II); *scale bars* 50 µm. **b** SFM and FCM cells also achieved osteogenic and adipogenic cytodifferentiation after 2 weeks of normoxic cultivation (Normo 2w), but failed to exhibit cytodifferentiation into either lineage after 2 weeks of hypoxic cultivation (3% O_2_ 2w). Notably, switching the culture condition from hypoxia for 2 weeks to normoxia for 2 weeks resulted in the development of ALZ-positive mineralized nodules and ORO-positive lipid droplets in SFM and FCM cultures, respectively (3% O_2_ 2w → Normo 2w). **c** Reverse-transcription polymerase chain reaction analysis revealed that 2-week-hypoxia-cultured PDLSCs that failed to undergo osteogenic (Os) and adipogenic (Ad) lineage differentiation exhibited higher expression of the stemness marker genes *Oct3/4*, *Nanog*, and *Sox2* (3% O_2_); after switching to normoxia, PDLSCs lost, or showed a lower expression of, stemness marker genes during cultivation for differentiation into both lineages (Normo)

To examine whether serum-free culture conditions enable the maintenance of stem cell characteristics and normal chromosomal stability under normoxia, phenotype, trilineage differentiation, and karyotype analyses were performed. In addition to the osteogenic/cementogenic, adipogenic, and chondrogenic lineages obtained by trilineage differentiation (Fig. 2a), a common MSC phenotype was indicated by flow cytometry, RT-PCR, and immunofluorescence (Supplemental Fig. 1). PDLSCs cultured in SFM or FCM exhibited normal karyotypes and diploid sets of chromosomes (2*n* = 46; data not shown). Collectively, these findings suggest that the serum-free culture environment does not affect PDLSC chromosomal stability, MSC phenotype, or multidifferentiation potential.

### Hypoxia does not alter the cell growth of PDLSCs cultured in SFM

Hypoxia facilitates the growth of cultured cells under conventional cultivation conditions in the presence of FBS [16, 17]. Therefore, we investigated whether hypoxia induced similar effects on PDLSC proliferation during cultivation in SFM. Hypoxia did not impact the fibroblastic cell morphology of PDLSCs cultured in SFM or FCM (Fig. 1a) including the significantly longer cell process length in SFM cells (Fig. 1b). However, hypoxia induced the active growth of FCM-PDLSCs as expected but not SFM-cultured PDLSCs, enhancing the proliferation of the former to levels comparable to those of SFM cells cultured under either O_2_ tension condition (Fig. 1c, d). Similar findings were observed by assessing PDT values, which were shorter (22.8, 22.5, and 22.7 h) for PDLSCs cultured in SFM under normoxia or hypoxia, and FCM under hypoxia, respectively, than for cells cultured in FCM under normoxia (30.9 h). Furthermore, the common MSC phenotype was observed following trilineage differentiation of SFM- and FCM-cultured PDLSCs (Supplemental Fig. 2 and the following section), suggesting that the hypoxic culture environment does not affect the MSC phenotype of these cells. Taken together, these findings suggest that hypoxic conditions are beneficial for ex vivo expansion of PDLSCs and maintenance of the stem cell phenotype during cultivation in FCM; however, hypoxia is not useful to accelerate the proliferation level of PDLSCs cultured in SFM.

### Hypoxia inhibits osteogenic and adipogenic cytodifferentiation, but maintains the multidifferentiation potential of PDLSCs

The cytodifferentiation of MSCs under conditions of various low-O_2_ tension (less than 8% O_2_) remains elusive. Moreover, successful MSC cytodifferentiation is dependent on the species, cell source, and cell isolation method [24–27]. We next examined the effect of hypoxia on the multidifferentiation potential of PDLSCs. Following culture in the respective induction media for differentiation into osteogenic/cementogenic, adipogenic, or chondrogenic lineages under hypoxic conditions for 4 weeks, no mineralized nodules or lipid vacuoles were observed in cultures maintained using SFM or FCM (Fig. 2a). This suggests that hypoxia inhibits PDLSC osteogenic and adipogenic cytodifferentiation.

To investigate whether PDLSCs cultured in hypoxia maintain their immature state and multidifferentiation potential, we firstly confirmed that osteogenic or adipogenic differentiation could be established under normoxic but not hypoxic conditions for 2 weeks (Fig. 2b). Notably, subsequently switching PDLSCs from hypoxia for 2 weeks to normoxia for 2 weeks allowed successful cytodifferentiation into both osteogenic and adipogenic lineages, as indicated by Alizarin red S-positive- and oil red O-positive-staining, respectively (Fig. 2b).

Furthermore, the expression of stemness marker-encoding genes including *Oct3/4*, *Nanog*, and *Sox2*, which are also involved in the maintenance of stemness [28, 29], was higher in the cytodifferentiation-inhibited PDLSCs cultured under hypoxia for 2 weeks than in the cytodifferentiated PDLSCs cultured under normoxia as ascertained by RT-PCR (Fig. 2c). This suggests that the former cells maintained their immature state with multidifferentiation potential. In contrast, hypoxia enhanced the chondrogenic differentiation of PDLSCs cultured in SFM or FCM (Fig. 2a), consistent with previous reports [30, 31]. Collectively, our data indicate that hypoxia does not affect PDLSC stemness, i.e., multidifferentiation potential, but enhances their chondrogenic differentiation, irrespective of serum use.

### PDLSCs are more susceptible to damage by extrinsic cytotoxic stimuli in SFM compared with FCM

**Fig. 3:**
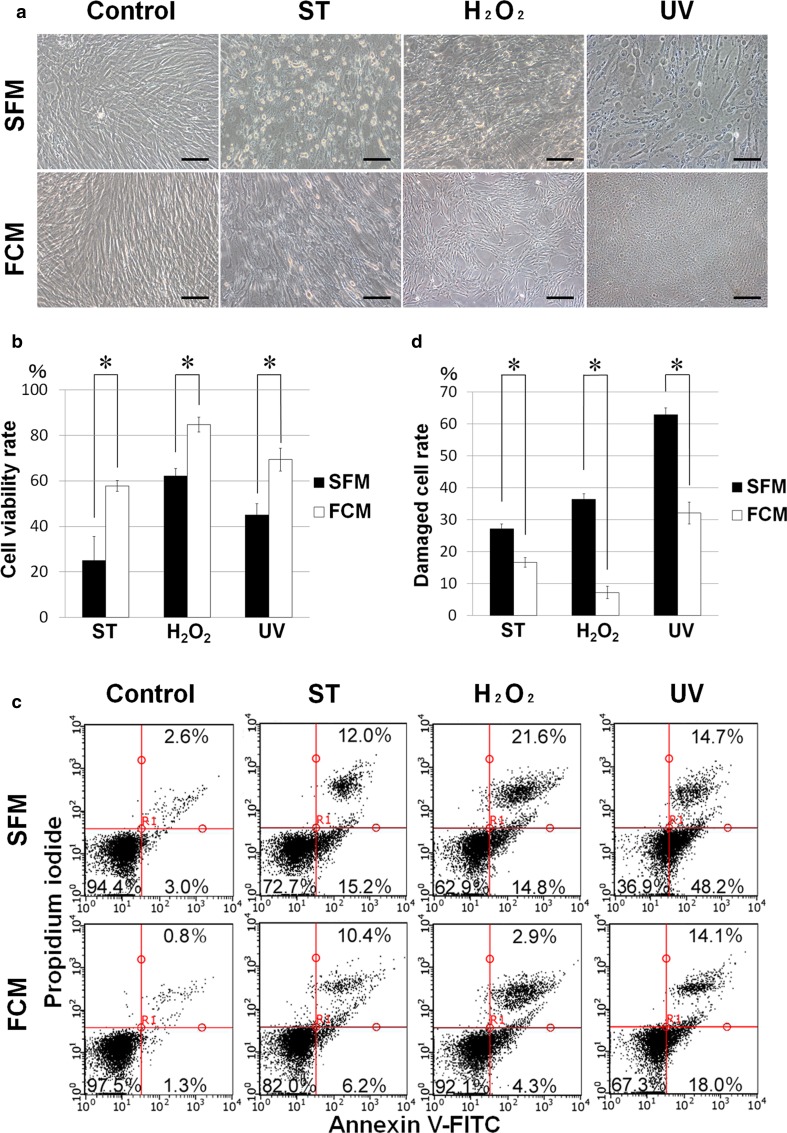
Cellular viability and stress/damage induced by extrinsic cytotoxicity under serum-free or FBS-containing culture condition. **a** Morphological alteration of the serum-free (SFM)- and FBS-containing (FCM)-cultured PDLSCs before (Control) and after cytotoxic treatments with staurosporine (ST), hydrogen peroxide (H_2_O_2_), and ultraviolet radiation (UV). All the cytotoxic stimuli-treated SFM cells showed damaged appearance with degenerative changes relative to the treated FCM cells; *scale bars* 100 µm. **b** The MTT assay revealed that viability of FCM cells was significantly higher than that of SFM cells after the cytotoxic treatments (*P < 0.01). **c** Flow cytometric analysis by Annexin V-FITC/PI staining showed that a greater amount of damaged cells, which were fractionated as both Annexin V-positive/PI-negative (early apoptotic cells) and Annexin V/PI-double positive (late apoptotic cells), were detected in SFM cultures compared with FCM cultures, following cytotoxic treatment. **d** Quantification of the flow cytometric analysis for damaged cells (*P < 0.01)

We next determined whether the presence of serum affects cell viability and cellular damage in PDLSCs exposed to various extrinsic stimuli. Cells were treated with widely known cytotoxic stimuli including H_2_O_2_ [32, 33], a reactive oxygen species that induces oxidative stress; UV radiation [34, 35]; or staurosporine [36, 37], which typically induces apoptotic death. Exposure to these agents caused degenerative morphologic changes in SFM- and FCM-cultured PDLSCs (Fig. 3a). Cell viability, as indicated by MTT assay, was significantly higher in FCM than in SFM cells (Fig. 3b) whereas the cellular stress/damage, as indicated by flow cytometry with Annexin V-FITC/PI staining, was significantly higher in SFM cells (Fig. 3c, d). Thus, the serum-free culture condition does not appear to exert protective effects against cytotoxicity on cultured cells exposed to extrinsic cytotoxic stimuli such as UV and chemical inducers of apoptosis.

### Ex vivo-expanded PDLSCs cultured in SFM generate cementum-like hard tissue in vivo

**Fig. 4:**
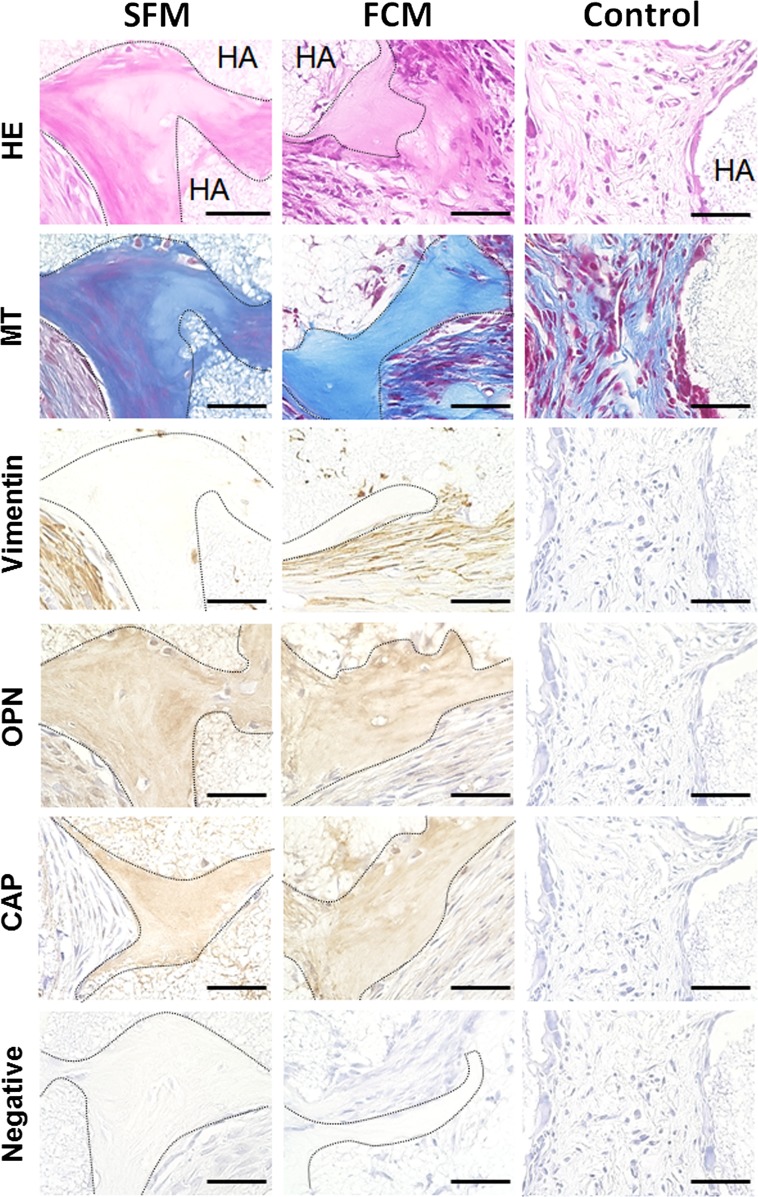
In vivo transplantation of ex vivo-expanded PDLSCs using serum-free- or FBS-containing medium; histological and immunohistochemical evaluation of cell/hydroxyapatite (HA)-scaffold constructs containing PDLSCs cultured in serum-free (SFM) or FBS-containing (FCM) medium in nude mice after subcutaneous transplantation. Hematoxylin and eosin (HE) and Masson’s trichrome (MT) staining indicated that the newly formed cementum hard tissue, which is indicated by the dashed line, was observed at the surface of HA-scaffold carrier (HA) in the SFM- and FCM-cell constructs. Immunoreactivity for the human-specific vimentin antibody was detected in the fibroblasts in connective tissue around the de novo hard tissue in both types of cell/scaffold constructs. Moreover, the hard tissue matrix was positively immunostained with antibodies against osteopontin (OPN) and cementum attachment protein (CAP), which are typical markers for cementum hard tissue. Neither hard tissue formation nor immunoreactivity for any antibodies was observed in the HA-scaffold alone (Control). The primary antibody was omitted during immunostaining (Negative); *scale bars* 100 µm

We next tested in vivo tissue formation by PDLSCs expanded using SFM or FCM, as shown in Fig. 4. Ex vivo-expanded PDLSC/HA-scaffold constructs were transplanted into the dorsal subcutaneous tissue of immunodeficient mice. Histological examination by hematoxylin and eosin and Masson’s trichrome staining showed that SFM- or FCM-cultured PDLSC/scaffold constructs but not HA scaffold alone (without cells) generated cementum-like hard tissue at 16 weeks post-implantation. Human-specific vimentin-positive cells, indicative of transplanted PDLSCs, were observed in connective tissue around the de novo hard tissue in both cell/scaffold constructs. Moreover, positive immunoreactivity for OPN, a non-collagenous protein present in mineralized tissues including the cementum [38], and CAP, a collagenous protein that promotes the attachment of mesenchymal stromal cells including cementoblasts [39], was observed within the newly formed hard tissue matrix but not within control transplant-associated fibroblasts and fibrous tissue. These findings indicate that PDLSCs expanded in SFM ex vivo maintained the capacity for de novo cementogenesis in vivo.

### Conclusions and perspectives

The present study indicates that commercially available SFM may facilitate the greater expansion of PDLSCs than conventional FCM. Moreover, SFM-expanded PDLSCs retained common MSC characteristics including the capacity for in vivo hard tissue formation. We further demonstrated that (1) hypoxia does not alter PDLSC growth under serum-free conditions, (2) hypoxia inhibits PDLSC osteogenic and adipogenic cytodifferentiation but enables maintenance of their multidifferentiation potential regardless of serum content, and (3) PDLSCs are likely damaged by a wide range of extrinsic cytotoxic stimuli under serum-free culture conditions.

Methods for the reliable and rapid expansion of primary cells from patients are necessary to acquire sufficient number of cells ex vivo for cell-based therapies. Commercially available SFM represents an optimal choice to facilitate cell growth but leaves cells susceptible to cell stress/damage induced by exposure to potentially cytotoxic stimuli during routine cell culture protocols. Alternatively, serum supplementation provides cytoprotective effects and enables reliable cell growth, which may be enhanced by hypoxic culture conditions to levels comparable to that in serum-free culture. Conversely, the hypoxic protocol is not necessary for MSC expansion in serum-free culture or for cytodifferentiation induction culture into osteogenic and adipogenic, but not chondrogenic lineages. Collectively, the present findings provide an evaluation of the practical feasibility of serum-free or serum-containing culture conditions for handling PDLSC cultures for clinical applications in regenerative medicine.

## Electronic supplementary material

Below is the link to the electronic supplementary material.

Supplemental Fig. 1 Gene expression and immunophenotyping of PDLSCs cultured in serum-free (SFM)- or FBS-containing (FCM)-culture medium under normoxia. **a** Flow cytometry indicated that the expression of widely known MSC markers (CD44, CD90, and CD105) was predominantly positive; in contrast, the expression of CD14 and CD34, which are hematopoietic cell markers, was essentially negative. Flow cytometry provided similar results for PDLSCs cultured with FCM under normoxia (data not shown). **b** The expression of genes encoding the typical markers for periodontal-lineage mesenchymal cells (*Vimentin*, *Type I collagen*, and *Runx2*), neural progenitor cells (*Nestin*), and pluripotent stem cells (*Nanog*, *Oct3/4*, and *Sox2*) was analyzed by reverse-transcription polymerase chain reaction. **c** Immunofluorescence indicated that PDLSCs cultured with SFM and FCM under normoxia were endogenously positive for neurogenic markers including nestin, neurofilament (NF)-200, and βIII-tubulin. These phenotypic profiles of PDLSCs are consistent with those described in our previous report [7]. *Scale bars* 50 µm (TIFF 20145 kb)

Supplemental Fig. 2 Gene expression and immunophenotyping of PDLSCs cultured in serum-free (SFM)- or FBS-containing (FCM)-culture medium under hypoxia. **a** Flow cytometry indicated that the expression of widely known MSC markers (CD44, CD90, and CD105) was predominantly positive; in contrast, the expression of CD14 and CD34, which are hematopoietic cell markers, was essentially negative. Similar results were obtained by flow cytometry for PDLSCs cultured with FCM under hypoxia (data not shown). **b** Reverse-transcription polymerase chain reaction analysis of the expression of genes encoding the typical markers for periodontal-lineage mesenchymal cells (*Vimentin*, *Type I collagen*, and *Runx2*), neural progenitor cells (*Nestin*), and pluripotent stem cells (*Nanog*, *Oct3/4*, and *Sox2*) in PDLSCs cultured in SFM and FCM under hypoxia. **c** Immunofluorescence indicated that PDLSCs cultured with SFM and FCM in hypoxia were endogenously positive for neurogenic markers including nestin, neurofilament (NF)-200, and βIII-tubulin. These phenotypic profiles of PDLSCs are consistent with those described in our previous report [7]. *Scale bars* 50 µm (TIFF 19871 kb)
