# Supplementary figures and images for: Practical methods for handling human periodontal ligament stem cells in serum-free and serum-containing culture conditions under hypoxia: implications for regenerative medicine
Source: Hum Cell. 2017 Feb 6;30(3):169–80. doi: 10.1007/s13577-017-0161-2 (PMC5486878; doi:10.1007/s13577-017-0161-2)

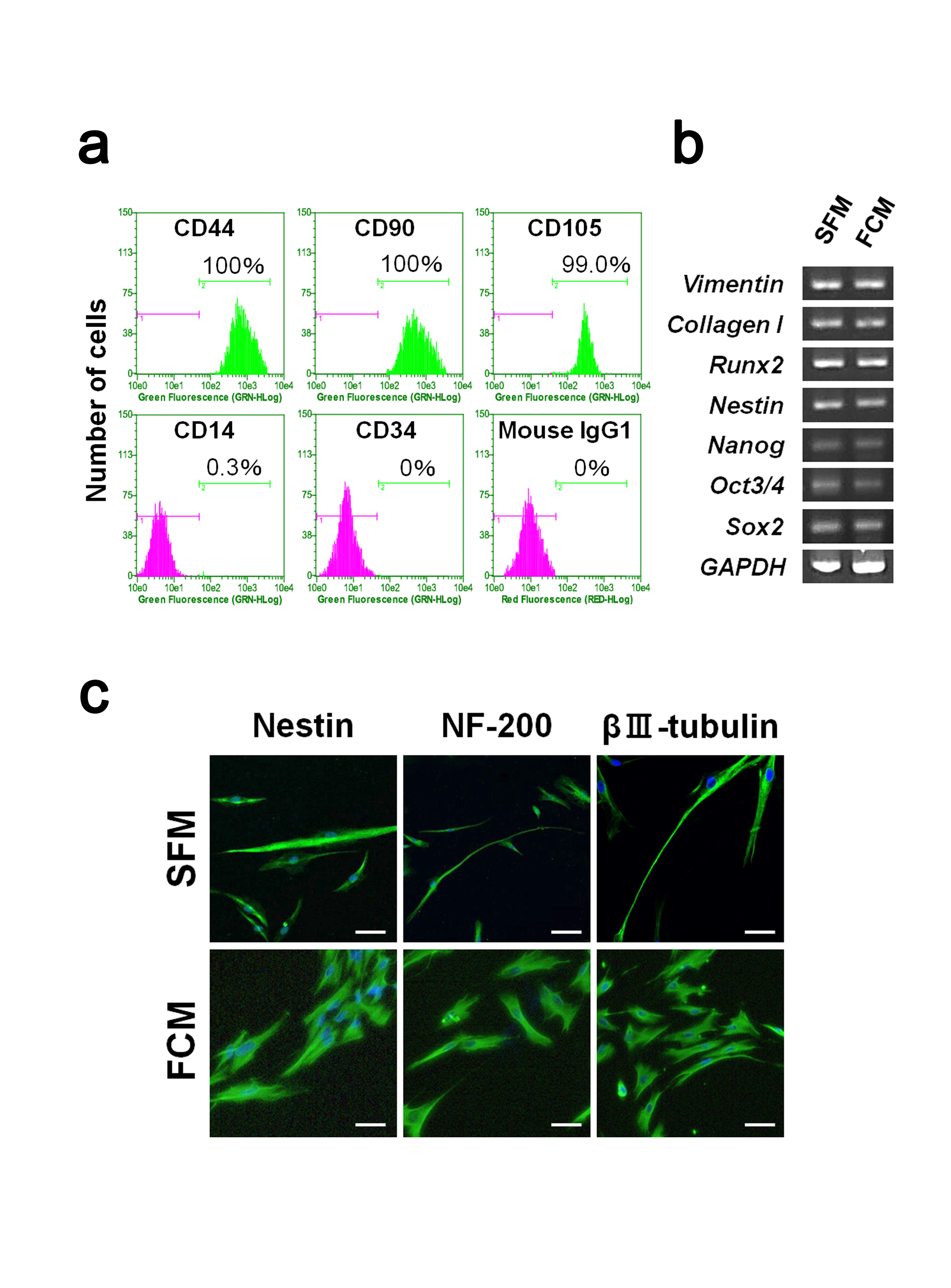

Supplement: Supplementary file 1 — Supplemental Fig. 1 Gene expression and immunophenotyping of PDLSCs cultured in serum-free (SFM)- or FBS-containing (FCM)-culture medium under normoxia. a Flow cytometry indicated that the expression of widely known MSC markers (CD44, CD90, and CD105) was predominantly positive; in contrast, the expression of CD14 and CD34, which are hematopoietic cell markers, was essentially negative. Flow cytometry provided similar results for PDLSCs cultured with FCM under normoxia (data not shown). b The expression of genes encoding the typical markers for periodontal-lineage mesenchymal cells (Vimentin, Type I collagen, and Runx2), neural progenitor cells (Nestin), and pluripotent stem cells (Nanog, Oct3/4, and Sox2) was analyzed by reverse-transcription polymerase chain reaction. c Immunofluorescence indicated that PDLSCs cultured with SFM and FCM under normoxia were endogenously positive for neurogenic markers including nestin, neurofilament (NF)-200, and βIII-tubulin. These phenotypic profiles of PDLSCs are consistent with those described in our previous report [7]. Scale bars 50 µm (TIFF 20145 kb) [file 13577_2017_161_MOESM1_ESM.tif]

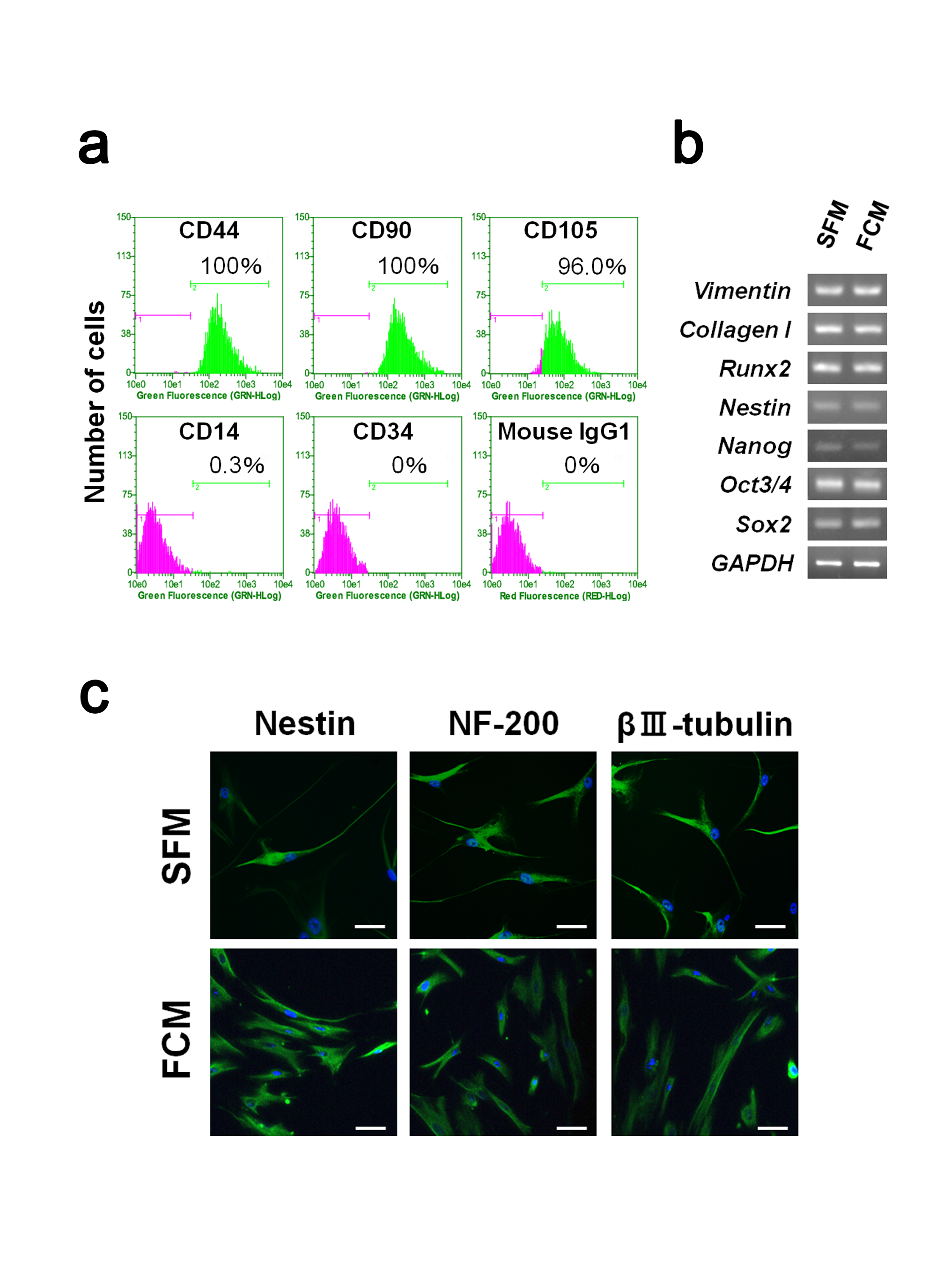

Supplement: Supplementary file 2 — Supplemental Fig. 2 Gene expression and immunophenotyping of PDLSCs cultured in serum-free (SFM)- or FBS-containing (FCM)-culture medium under hypoxia. a Flow cytometry indicated that the expression of widely known MSC markers (CD44, CD90, and CD105) was predominantly positive; in contrast, the expression of CD14 and CD34, which are hematopoietic cell markers, was essentially negative. Similar results were obtained by flow cytometry for PDLSCs cultured with FCM under hypoxia (data not shown). b Reverse-transcription polymerase chain reaction analysis of the expression of genes encoding the typical markers for periodontal-lineage mesenchymal cells (Vimentin, Type I collagen, and Runx2), neural progenitor cells (Nestin), and pluripotent stem cells (Nanog, Oct3/4, and Sox2) in PDLSCs cultured in SFM and FCM under hypoxia. c Immunofluorescence indicated that PDLSCs cultured with SFM and FCM in hypoxia were endogenously positive for neurogenic markers including nestin, neurofilament (NF)-200, and βIII-tubulin. These phenotypic profiles of PDLSCs are consistent with those described in our previous report [7]. Scale bars 50 µm (TIFF 19871 kb) [file 13577_2017_161_MOESM2_ESM.tif]
